# Sex: Not all that it’s cracked up to be?

**DOI:** 10.1371/journal.pgen.1007160

**Published:** 2018-02-22

**Authors:** Sebastian Eves-van den Akker, John T. Jones

**Affiliations:** 1 Biological Chemistry, John Innes Centre, Norwich Research Park, Norwich, United Kingdom; 2 School of Life Sciences, University of Dundee, Dundee, United Kingdom; 3 Cell and Molecular Sciences Group, Dundee Effector Consortium, James Hutton Institute, Dundee, United Kingdom; 4 School of Biology, University of St Andrews, North Haugh, St Andrews, United Kingdom; National Institute of Genetics, JAPAN

While sexual reproduction is generally thought to be, evolutionarily speaking, a good idea, there are a small number of organisms that are testament to the contrary. The root-knot nematodes *Meloidogyne incognita*, *M*. *javanica*, and *M*. *arenaria* reproduce clonally using mitotic parthenogenesis but have a broader host range, a wider geographical distribution, and a greater agricultural impact than their sexual relatives (Fig 1) [1]. Remarkably, some of these species even have the ability to overcome host resistance [2], suggesting a mechanism for adaptation in the absence of sex. The genetic basis of this plasticity, both in terms of host range and adaptability, is not fully understood. Previous genome sequencing of *Meloidogyne* has shown that the genome of one of these species, *M*. *incognita*, is polyploid [3], most likely as a result of hybridisation (allopolyploid), with a further study suggesting that *M*. *incognita* may be the result of multiple additive hybridisation events: a hybrid of a hybrid [4].

**Fig 1:**
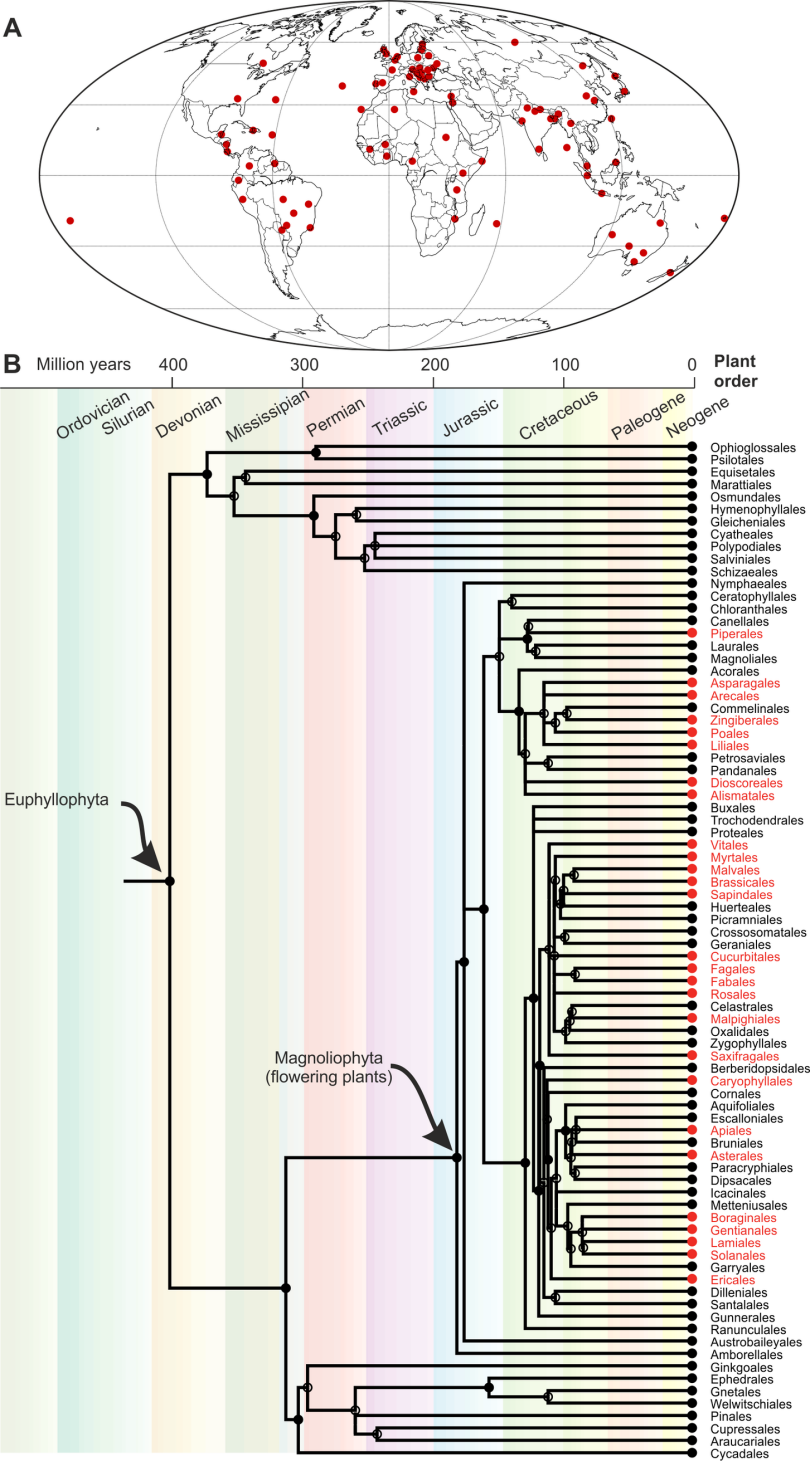
Geographical distribution and host range of *M*. *incognita*. Information from the Cookies on Invasive Species Compendium (CABI) data sheet (https://www.cabi.org/isc/datasheet/33245; accessed 23 November 2017). All data are nonexhaustive; the absence of evidence is not evidence of absence. (A) Geographical distribution. (B) Partial phylogenetic time tree of plants (timetree.org; accessed 23 November 2017). Red indicates orders with at least 1, but often more, host species. The extraordinarily broad host range of *M*. *incognita* covers most orders in the flowering plants (Magnoliophyta) but is apparently restricted to flowering plants—although this may reflect testing bias.

To understand the impact of allopolyploidy on plasticity, Blanc-Mathieu et al. [5] resequenced the *M*. *incognota* genome and transcriptome (4 life stages) and sequenced the genomes of 2 related mitotic parthenogens, *M*. *javanica* and *M*. *arenaria*. These data were compared to existing assemblies of the meiotic parthenogen *M*. *floridensis* and the facultative sexually reproducing *M*. *hapla*.

## Bewildering complexity

As expected, 89% of coding sequences in the sexual *M*. *hapla* align to a single locus in the genome (>95% identity across ≥2/3 of length), while in contrast, >85% of the coding sequences in the parthenogens *M*. *incognita*, *M*. *javanica*, and *M*. *arenaria* align to at least 2 loci, but often more. Assuming the number of alignments is a rough proxy for copy number, *M*. *incognita* and *M*. *javanica* have more genes with at least 3 copies than any other copy number. For *M*. *arenaria*, this phenomenon is further pronounced: more genes have at least 4 copies than any other copy number. Blanc-Mathieu et al. [5] show that multicopy genome regions have different evolutionary histories and thus support multiple different hybridisation events.

Following a sudden gain in ploidy, one of each gene copy can often be lost, but this does not seem to have been the case yet in relatively “young” *Meloidogyne* species; only 4%–6% of ancestral collinear genes seem to have been lost after the whole genome duplication event, and most of the coding potential of each genome has therefore been retained. The end result is that the coding potential is enormous: up to 100,000 predicted loci. In addition, compared to other nematode genomes, those of the mitotic parthenogens also contain an extremely high proportion of transposable elements (up to 50% of the genome). Given that transposable element predictions wholly include 27%–30% of predicted protein-coding loci, the authors suggest that this may have an adaptive impact, although recent phylum-wide analysis suggests that drift and purifying selection, not life history, determine transposable element evolution in Nematoda [6]. However, several mechanisms were identified that may allow a significant adaptive impact: (1) multicopy loci are on average approximately 8% sequence divergent; (2) genes in the majority of the expressed homologous gene pairs were assigned to different expression clusters (64%); and (3) gene pairs showing signs of diversifying selection are more often present in different differential expression clusters to one another than those with signs of purifying selection.

## The stage is set

It will now be possible to explore how the sequence divergence and apparent functional divergence of homologous gene copies relate to the parasitic lifestyle. Analysis of differential expression—and, indeed, differential alternative splicing of homologous gene copies across the host range of *M*. *incognita* may provide a tangible link between the enormous apparent coding potential and host range. It is worth noting that, assuming the parents of the hybridisation events that gave rise to these *Meloidogyne* were not homozygous at every allele, an additional level of allelic variation is yet to be explored. It is not clear to what extent mitotic recombination may have contributed to introducing heterogeneity, removing deleterious mutations, and fixing adaptive mutations immediately after the hybridisation event or more recently. Haplotype sequencing may provide an answer to some of these questions in the future.

One question that needs to be resolved is to what extent the genomes sequenced to date [5] are representative of the species. Following the loss of sexual reproduction, there is presumably no genetic exchange between individuals, and thus, in terms of gene flow, each individual can be conceptually regarded as a separate species. Given that recombination has clearly taken place in the genomes of the mitotic parthenogens [5], comparing the genomes of isolates that have reproduced under different selection pressures would be fascinating. Because of the gene flow barrier imposed by abandoning sex, these need not necessarily be geographically distinct; nevertheless, it would be interesting to explore the impact of prolonged exposure to different hosts and environments on genome evolution. Sequencing several geographically separated isolates may also help to explain the apparent contradiction of a relatively recent evolutionary origin but worldwide distribution of these species (Fig 1).

Finally, it is thought that the ability of a parasite to manipulate its host is due to the activities of effectors: secreted molecules that are delivered into the host during infection. The effector complement of the tropical root-knot nematodes was not explored in the present study. Considering the often unusual genome evolution of effectors (reviewed in [7]), analysing the arrangement, expression, regulation, and diversity of effectors in the polyploid *Meloidogyne* would be intriguing. It has been suggested that the success of *M*. *incognita* is consistent with the phenomenon of transgressive segregation [4]: hybrids often outperform their parents for a given phenotype. How transgressive segregation scales with multiple (additive) hybridisation events is unclear, but this may explain a host range that encompasses almost every flowering plant.
